# Feasibility of MR-guided ultrahypofractionated radiotherapy in 5, 2 or 1 fractions for prostate cancer

**DOI:** 10.1016/j.ctro.2020.10.005

**Published:** 2020-10-27

**Authors:** Jonathan Mohajer, Alex Dunlop, Adam Mitchell, Edmund Goodwin, Simeon Nill, Uwe Oelfke, Alison Tree

**Affiliations:** a Joint Department of Physics, The Institute of Cancer Research and The Royal Marsden NHS Foundation Trust, 15 Cotswold Road, London, Surrey SM2 5NG, UK; b Department of Urology, The Royal Marsden NHS Foundation Trust, 15 Cotswold Road, London, Surrey SM2 5NG, UK; c The Institute of Cancer Research, 15 Cotswold Road, London, Surrey SM2 5NG, UK

**Keywords:** MR-linac, Prostate, SBRT, Ultrahypofractionation, MRgRT, Extreme hypofractionation

## Abstract

The drive towards hypofractionated prostate radiotherapy is motivated by a low alpha/beta ratio for prostate cancer (1 to 3 Gy) compared to surrounding organs at risk, implying an improved therapeutic ratio with increasing dose per fraction. Early evidence from studies of ultrahypofractionated (UHF) prostate HDR brachytherapy has shown good tolerability in terms of normal tissue toxicities and clinical outcomes similar to conventional fractionation schedules.

MR-guided stereotactic body radiotherapy (SBRT) with online plan adaptation and real-time tumour imaging may enable UHF doses to be delivered to the prostate safely, without the invasiveness of brachytherapy. The feasibility of UHF prostate treatment planning for the Unity MR-Linac (MRL, Elekta AB, Stockholm) was investigated for target prescriptions and planning constraints derived from the HDR brachytherapy and SBRT literature.

Monaco 5.40 (Elekta) was used to generate MRL step-and-shoot IMRT plans for three dose fractionation protocols (5, 2 and 1 fractions), for ten randomly selected previously treated prostate cancer patients. Of the ten plans per UHF scheme, all clinical goals were met in all cases for 5 fractions, and in six cases for both 2 and 1 fraction schemes. PTV D95% was compromised by up to 6.4% and 3.9% of the associated target dose for 2 and 1 fraction plans respectively. There were two cases of PTV D95% compromise greater than a 5% dose decrease for the 2 fraction plans. The study suggests feasibility of the UHF treatment planning approaches if combined with real-time motion mitigation strategies.

## Highlights

• Deliverable plans for 1 and 2 fraction SBRT can be created for the Unity MR-Linac.
• Dose constraints and objectives from HDR and SBRT clinical studies were applied.
• In 40% of 1 and 2 fraction plans target doses were compromised to meet OAR goals.

## 1 Introduction

Over the last decade, stimulated by the accumulating laboratory and clinical evidence supporting a low alpha/beta ratio for prostate cancer, many trials of hypofractionation in prostate radiotherapy have been completed. Initially testing moderate hypofractionation (dose per fraction 2.5–3.4 Gy), these trials have shown that these regimens are non-inferior to traditional 2 Gy per fraction schedules [1], [2], [3], [4]. Subsequent trials have tested ultrahypofractionation (UHF), initially testing seven [5] then five fractions [6] at 6.1 to 7.25 Gy per fraction. To date, all studies have shown equivalence of hypofractionation.

More extreme hypofractionation has been tested with high dose rate (HDR) brachytherapy, down to 3, 2 or even a single fraction [7], [8], [9] with limited experience with similar fractionations delivered with external beam radiotherapy [10]. However, recently reported poor PSA control rates with single fraction HDR have significantly tempered enthusiasm for this approach [11], [12], [13].

Magnetic resonance imaging (MRI), done at biopsy, frequently can locate the dominant site of tumour. This lesion is the area within the prostate most likely to result in treatment failure [14] hence it is logical to explore focal dose escalation rather than whole gland dose escalation, which is known to increase toxicity rates. The focal boost concept has been tested in the FLAME [15], BIOPROP [16] and DELINEATE [17] trials. Data indicates that focal boosting can be achieved without a toxicity penalty. Biochemical outcomes are expected shortly.

MR-guided radiotherapy has become a reality within the last few years [18], [19], [20]. Whilst stereotactic body radiotherapy (SBRT) can be administered using systems such as CyberKnife and C-arm linear accelerators, online MR-guidance provides excellent soft tissue contrast prior to, and during, radiotherapy delivery, increasing accuracy. In addition, it allows a plan to be created whilst the patient is on the treatment couch, allowing an improved match of dosimetry to patient anatomy. Furthermore, real-time MR-guided plan adaptation strategies have been developed to mitigate against intrafractional anatomical motion [21], [22]. This is therefore the ideal system to deliver UHF radiotherapy, such that dose can be maximised to the target and minimised to the organs at risk, based on up to date anatomical information. At present, only fixed field intensity modulated radiotherapy (IMRT) can be delivered on the Unity MR-Linac (MRL, Elekta AB, Stockholm) and dose rate is limited by the extended focus-to-skin distance and cryostat transmission, hence the practicalities of delivering UHF with MR-guided radiotherapy require further investigation.

This project sought to examine whether 5, 2 and 1 fraction SBRT can be planned for the Unity MRL whilst respecting dose constraints established by HDR or feasibility studies in external beam radiotherapy.

## 2 Methods

### 2.1 Patient selection

Ten CT scans and structure sets (target and organ at risk delineations) of consented patients previously treated at our centre as part of the DELINEATE trial (ISCTRN 04483921; dose escalation to intraprostatic tumour nodules in localised prostate cancer) were randomly selected. Patients were included if the clinical target volume (CTV) volume was below 50 cc and dominant intraprostatic lesion (DIL) volume less than one quarter of the CTV volume.

### 2.2 Planning dose constraints for hypofractionation schemes

PACE (Prostate Advances in Comparative Evidence, NCT01584258) SBRT low to intermediate risk planning constraints were applied to 5 fraction plans, which was used as the standard comparison here. Dose constraints for the 2 and 1 fraction treatments were taken from published HDR series with toxicity outcomes or, by preference, SBRT clinical series where available (Table 1, Table 2). Target doses were taken from clinical studies and the equivalent dose in 2 Gy fractions (EQD2) of these regimens is compared in Table 3, assuming low alpha/beta ratios of 1.5 Gy or 3 Gy.

**Table 1:** Summary of treatment planning dosimetric constraints and volume definitions for radiotherapy delivered in two fractions. Dosimetric constraints apply to the sum over the total course. Prescription conditions are indicated by asterisks. Abbreviations: high dose-rate brachytherapy (HDR), stereotactic body radiotherapy (SBRT), planning target volume (PTV), clinical target volume (CTV), gross tumour volume (GTV).

Region of interest		Hoskin et al. [7], [24] (HDR)	Ghilezan et al. [25] (HDR)	Jawad et al. [26] (HDR)	Morton et al. [8] (HDR)	Alayed et al. [10] (SBRT)	Present study (SBRT)
PTV Prostate	*Constraints*	min peripheral dose = 26 Gy*	V27 Gy > 97%* V33.75 Gy < 60% V40.5 Gy < 30%	V27 Gy > 95%* V33.75 Gy < 60% V40.5 Gy < 30%	V27 Gy > 95%* V40.5 Gy < 35% V54 Gy < 12%	D99% (CTV) = 26 Gy*	D95% ≥ 24 Gy* D98% ≥ 22.8 Gy D2% < 29.7 Gy max dose (excl. GTV) < 30 Gy
	*Volume definition*	CTV + 3 mm uniform expansion, clipped at rectum	CTV, no expansion	CTV, no expansion	CTV, no expansion	CTV + 3 mm uniform expansion	CTV + 2 mm uniform expansion
GTV Prostate_Boost	*Constraints*	–	–	–	–	–	D95% ≥ 27 Gy* max dose < 33.75 Gy
	*Volume definition*	–	–	–	–	–	Dominant intraprostatic lesion, no expansion
Rectum	*Constraints*	D0.25 cc < 25 Gy D2 cc < 20 Gy	max dose < 19.58 Gy	V20.25 Gy < 1%	max dose < 24.3 Gy V21.6 Gy < 0.2 cc	V20.8 Gy < 1 cc V17.6 Gy < 4 cc V13 Gy < 7 cc	V20.8 Gy < 1 cc V17.6 Gy < 4 cc V13 Gy < 7 cc
Bladder	*Constraints*	–	–	–	–	V20.8 Gy < 5 cc V14.6 Gy < 15 cc	V20.8 Gy < 5 cc V14.6 Gy < 15 cc
Urethra	*Constraints*	max dose < 30 Gy D30% < 28.5 Gy	V27 Gy < 10%	V31.05 Gy < 1% V27 Gy < 95% (pref. 90%)	max dose < 32.4 Gy D10% < 31.05 Gy	–	D10% < 27 Gy
Femoral heads	*Constraints*	–	–	–	–	V14 Gy < 10 cc	V14 Gy < 10 cc

**Table 2:** Summary of treatment planning dosimetric constraints and volume definitions for radiotherapy delivered in a single treatment. Prescription conditions are indicated by asterisks. Abbreviations: high dose-rate brachytherapy (HDR), stereotactic body radiotherapy (SBRT).

Region of interest		Hoskin et al. [7], [24] (HDR)	Krauss et al. [27] (HDR)	Morton et al. [8] (HDR)	Gomez-Itturiaga et al. [28] (HDR)	Present study (SBRT)
PTV Prostate	*Constraints*	min peripheral dose = 19 Gy*	V19 Gy > 95%* V23.75 Gy < 60%	V19 Gy > 95%* V28.5 Gy < 35% V38 Gy < 12%	min peripheral dose = 19 Gy* V19 Gy > 95% V28.5 Gy = 25 to 35% V38 Gy < 8%	D95% ≥ 19 Gy* D99% ≥ 18.05 Gy max dose (excl. GTV) < 23.75 Gy
	*Volume definition*	CTV + 3 mm uniform expansion, clipped at rectum	CTV, no expansion	CTV, no expansion	Not reported	CTV + 2 mm uniform expansion
GTV Prostate_Boost	*Constraints*	–	–	–	–	D95% ≥ 21 Gy* D99% > 19.95 Gy max dose < 26.25 Gy
	*Volume definition*	–	–	–	–	Dominant intraprostatic lesion, no expansion
Rectum	*Constraints*	D0.04 cc < 19 Gy D2 cc < 15 Gy	max dose < 13.8 Gy	max dose < 17.1 Gy D15.2 Gy < 0.2 cc	D1 cc < 11.4 Gy	D0.04 cc < 19 Gy D2 cc < 15 Gy D50% < 12 Gy
Bladder	*Constraints*	–	–	–	–	D50% < 12 Gy
Urethra	*Constraints*	max dose < 28.5 Gy D10% < 22 Gy D30% < 20.8 Gy	V20.9 Gy < 10%	max dose < 22.8 Gy D10% < 21.85 Gy	max dose < 20.9 Gy	max dose < 22.8 Gy D10% < 21 Gy

**Table 3:** Comparison of equivalent dose in 2 Gy fractions (EQD2) for target doses used in this study. Abbreviations: dominant intraprostatic lesion (DIL), planning target volume (PTV). *In the 5 fraction scheme, 40 Gy is prescribed to the whole prostate clinical target volume as opposed to DIL.

Number of fractions	Prostate PTV dose (Gy)	DIL dose (Gy)	EQD2 (Gy)	
			α/β = 1.5 Gy	α/β = 3 Gy
1	19		111	84
		21	135	101
2	24		93	72
		27	116	89
5	36.25		91	74
		40*	109	88

### 2.3 Treatment planning

Monaco 5.40.01 was used to generate Unity MRL step-and-shoot intensity-modulated radiotherapy (IMRT) plans for three dose fractionation protocols, testing 5, 2 and 1 fraction plans for the ten patients.

**Fig. 1:**
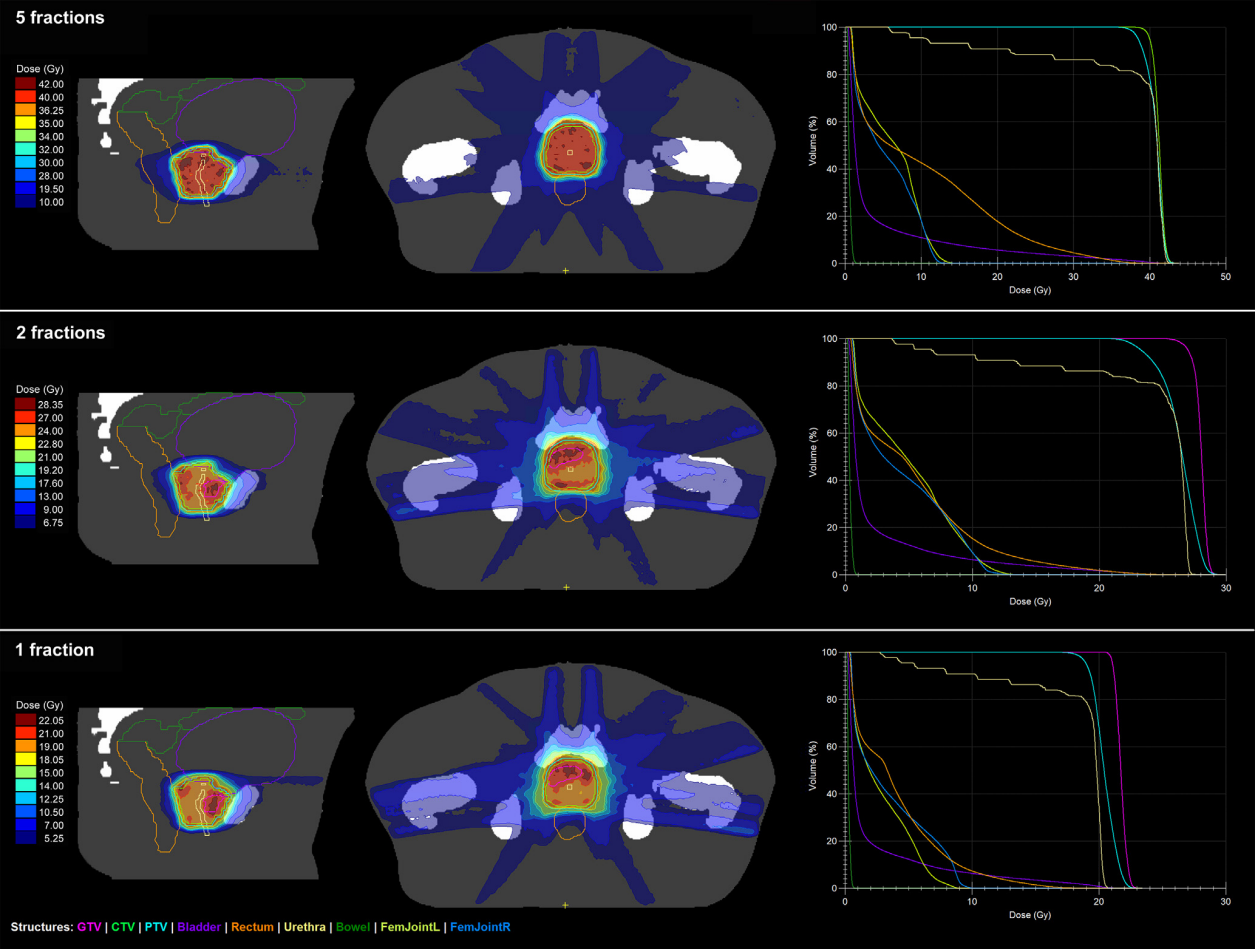
Example Unity MR-Linac stereotactic body radiotherapy dose distributions for plans created for one patient using the three fractionation schemes. Synthetic CT data is displayed, illustrating the bulk density assignment used for dose calculation.

CT images were acquired with a slice thickness of 1.5 mm. The DIL volume was designated the gross tumour volume (GTV), the prostate including proximal 1 cm seminal vesicles the CTV and the planning target volume (PTV) defined as a uniform expansion of the CTV by 2 mm (see Fig. 1). GTV delineation was based upon multi-parametric MRI data registered to the planning CT [17]. The MR sequence favoured for urethra delineation was a standard T2 diagnostic sequence, with particular attention paid to the urethral contour on the sagittal image.

In order to simulate the MR-Linac online treatment planning workflow, treatment planning was performed with patient-specific bulk relative electron density (rED) values assigned to three regions of interest (ROIs); the bones, CTV and patient external. The rED values assigned to these regions were calculated using the CT Hounsfield unit to rED lookup table, taken as the average rED over a sub-region of the ROI centred on the CTV, to include CT slices 5 mm above and below the CTV. Restriction of the superior-inferior extent of the CT data for rED sampling was performed to obtain reasonably accurate bulk densities for the bones and patient external (excluding bones and CTV) within the irradiated volume. An assessment of the dosimetric impact of the bulk density override strategy was performed (see Appendix A).

Monaco IMRT optimisation and dose calculation settings are given in Table 4. These settings were selected to facilitate online plan optimisation (subsequent to contouring) in less than six minutes, and to restrict the MU and number of segments such that treatment delivery times may be restricted as far as possible without significant detriment to plan quality. All IMRT plans used 9 beams at gantry angles 0°, 50°, 75°, 100°, 150°, 210°, 260°, 285° and 310°. Optimisation prioritised OAR objectives over target objectives.

**Table 4:** Monaco treatment plan settings.

Calculation settings	*Dose engine*	GPUMCD
	*Dose quantity*	Dose to medium
	*Grid spacing*	0.2 cm isotropic
	*Statistical uncertainty*	1.5% per calculation
	*Static magnetic field*	1.5 T

IMRT parameters	*Target margin*	Very tight (0 – 1 mm)
	*Avoidance margin*	Very tight (0 – 1 mm)

Segmentation settings	*Segment shape optimisation (SSO) loops*	5
	*Maximum segments*	65
	*Minimum segment area*	4 cm^2^
	*Minimum segment MU*	4 MU

Treatment plan dose distributions were evaluated against the dose constraints presented in Table 1, Table 2. Furthermore, conformity was assessed using the PTV conformation number (CN) [23]:

$$\mathrm{CN}=\frac{TV_{\mathrm{RI}}}{\mathrm{TV}}∙\frac{TV_{\mathrm{RI}}}{V_{\mathrm{RI}}}$$

where $TV_{\mathrm{RI}}$ is the structure volume covered by the dose of interest, TV is the structure volume and $V_{\mathrm{RI}}$ is the total volume of the dose of interest.

### 2.4 Treatment plan delivery verification

A prototype PTW Octavius 4D MR with Octavius 1500 detector array (PTW Freiburg GmbH) was used for treatment plan delivery verification. The centre of the detector array was aligned to the radiation isocentre by way of a Perspex jig fixed to the MR-Linac bridge.

PTW Verisoft v7.2 software was used to perform a gamma evaluation of the measured dose against the Monaco calculated dose for 3 plans in each of the prescription schemes. Gamma evaluation criteria were consistent with those used for clinical treatments assessed using the PTW Octavius 4D MR; 2% dose difference (global), 2 mm distance to agreement. Measured and calculated dose distributions were normalised in reference to the maximum calculated dose. Gamma pass rate was defined as the percentage of all voxels evaluated in the measured dose distribution where γ < 1.

## 3 Results

**Fig. 2:**
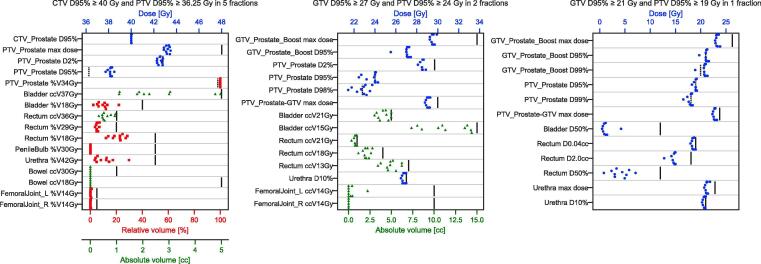
Clinical goals for each of the ten plans generated for each of the UHF schemes. Solid vertical lines indicate a clinical goal maximum threshold, with a dashed line indicating a minimum threshold.

Of the ten plans per UHF scheme, all clinical goals were met in all cases for 5 fractions, and in six cases for both 2 and 1 fraction schemes (see Fig. 2). PTV D95% was compromised by up to 6.4% and 3.9% of the associated target dose for 2 and 1 fraction plans respectively, corresponding to doses of 1.54 Gy and 0.74 Gy. There were two cases of PTV D95% compromise greater than a 5% dose decrease for the 2 fraction plans.

PTV CN medians and ranges were: 5 fractions 0.84 (0.81 – 0.86); 2 fractions 0.83 (0.77 – 0.89); 1 fraction 0.83 (0.78 – 0.86).

Mean and standard deviation treatment delivery times were: 5 fractions (7.9 ± 0.5 min); 2 fractions (11.5 ± 0.9 min); 1 fraction (16.0 ± 1.6 min).

Treatment plan delivery verification mean gamma pass rates for the three plans measured for each of the UHF schemes were: 5 fractions (95.9%); 2 fractions (94.4%); 1 fraction (93.4%).

## 4 Discussion

The study represents an initial step towards clinical implementation of MR-guided UHF prostate treatments. In just under half of 2 and 1 fraction plans target doses were compromised to meet OAR constraints; in two of ten 2 fraction plans PTV D95% was compromised significantly (i.e. underdosed by>5% with respect to the prescription).

In response to the limited clinical OAR toxicity data associated with UHF prostate radiotherapy available, the most conservative planning constraints utilised in HDR brachytherapy monotherapy studies [7], [8], [24], [25], [26], [27], [28], along with one UHF SBRT study [10] were applied. Gastrointestinal and genitourinary toxicities in these studies were generally mild, with toxicities greater than grade 2 (variously according to RTOG and CTCAE criteria) very rarely observed. Whilst single fraction OAR dosimetric constraints are well-tolerated, evidence published since initiating the present study has shown poorer efficacy for a single dose of 19 Gy to the prostate, particularly for intermediate- and high-risk patients [11], [12], therefore is not currently recommended for clinical implementation. We posit that the dosimetric constraints utilised in the present study for two fraction prostate SBRT are well-supported by the available clinical evidence and represent a suitable framework for future clinical trials.

The PTV in the present study was a 2 mm uniform expansion of the CTV prostate. Online plan adaptation, in particular where the CTV is re-contoured online, eliminates interfractional geometric uncertainty. Menten et al. [29] analysed the intrafractional motion of the prostate using template matching in cine-MR for five Unity MR-Linac prostate cancer patients treated with 60 Gy in 20 fractions. For the duration of treatment delivery (5.5 ± 0.8 min), mean and standard deviation CTV positional shifts of ﻿0.0 ± 0.8 mm (posterior direction) and 0.1 ± 0.9 mm (caudal direction) were reported. More significant motion was observed by de Muinck Keizer et al. [30] over a period of ten minutes; tracking of implanted gold fiducial markers in cine-MR acquisitions yielded mean and standard deviation centre of mass translations ﻿0.0 ± 0.8 mm (lateral), 1.0 ± 1.9 mm (posterior direction) and 0.9 ± 2.0 mm (caudal direction). Cumulative absolute centre-of-mass displacement exceeded 2 mm in 72% of cases over a period of 10 min, compared with 55% at 7 min. Since the five, two and one fraction treatment plans typically exceeded a seven-minute delivery in the present study, the tendency for both systematic and random components of intrafractional motion to increase with time [30] suggests that real-time adaptation, such as MLC tracking or gating, may be necessary in order for a 2 mm PTV margin to be realised. Dynamic tumour tracking strategies applicable to the MR-Linac are associated with system root mean square geometric errors of 1.1 mm for target velocities ≤ 20 mm s^−1^ [31]. Further work is required to measure such uncertainties for the Elekta Unity system utilising cine-MR motion monitoring.

In addition to patient motion occurring during treatment delivery, there is a likelihood of patient motion during the various stages of online plan adaptation subsequent to acquisition of the daily planning MR. Where patient motion can be adequately approximated by a translational offset, a relatively quick ‘adapt to position’ workflow may be utilised to compensate. This effect is analogous to a couch shift on a C-arm linac. Significant changes in anatomical morphology over this period are rare but would present a greater challenge, potentially necessitating re-contouring and re-planning again.

The feasibility of online MR-guided adaptive radiotherapy is dependent upon treatment durations (i.e. total time of the patient immobilised on the treatment couch) which are well-tolerated by patients. Our experience of treating prostate cancer patients in the PRISM trial (Prostate Radiotherapy Integrated with Simultaneous MRI, NCT03658525) showed that 27 patients of 28 treated did not request to interrupt the online planning workflow. For the three UHF SBRT schemes investigated, optimisation and calculation settings were selected to achieve an acceptable balance between plan quality (encompassing dosimetric precision and plan optimality) and speed (in terms of both plan optimisation and delivery times). Based upon our experience of the PRISM trial, it is anticipated that treatment sessions (including patient set-up, MR imaging, contour propagation and editing, plan generation, plan checking and treatment delivery) of less than one hour may be realised for the UHF treatment planning approaches presented. Treatment plan delivery verification results showed a high level of agreement between planned and measured radiation doses, affirming the clinical suitability of the calculation and segmentation settings employed.

Given the strict OAR sparing employed in the UHF SBRT planning strategies presented, it is evident that physical optimisation of the patient’s anatomy at each treatment session is critical to enabling maximal target coverage. The use of hydrogel rectal spacers to temporarily enlarge the perirectal space has been associated with low toxicity in the context of prostate SBRT [32], [33]. Optimal bladder filling for MR-Linac prostate radiotherapy relies upon a careful balance between the presentation of a sufficiently full bladder to displace the small bowel superiorly and the patient’s ability to comfortably hold their bladder for the duration of treatment [34]. Pre-treatment MR simulation may assist in the refinement of the drinking schedule to best achieve such optimal bladder filling during treatment. Urinary catheterisation as a means of retaining constant bladder filling provides an alternative approach.

UHF prostate SBRT on the Unity MR-Linac involves the presence of a strong static magnetic field during treatment delivery. The application of tissue bulk densities to regions of interest to facilitate dose calculation on MR images has the potential to introduce significant dosimetric errors in scenarios such as the presence of rectal gas proximal to the treated region [35]. The electron return effect gives rise to dose enhancement at air-tissue interfaces under such conditions [36], however if these regions are not specified during plan optimisation, their impact would not be mitigated. It has been suggested that the dosimetric impact of unplanned rectal gas on prostate MRL IMRT treatment plans may be sufficient to warrant intervention in the context of UHF SBRT [35]. Whilst delineation and bulk density assignment of air regions and synthetic CT generation from MRI represent two possible approaches to achieving acceptable dose calculation accuracy, the mobility of such air regions is very difficult to account for. A better approach would be to use patient strategies to expel excess gas prior to starting the workflow, if possible.

Whilst MRL online imaging is well-suited to the delineation of many features of pelvic anatomy relevant to prostate radiotherapy, DIL and urethra visibility are likely to be suboptimal in many cases. As such, we propose that these two structures are propagated to the daily MR from imaging data previously delineated, via soft-tissue based rigid registration.

Single HDR treatments have been proven to result in poorer biochemical relapse-free survival than would be achieved with standard fractionation [11], [12]. Therefore, at present, single fraction SBRT no longer presents a promising line of study. In contrast, two fraction regimens appear to be more efficacious and just as well tolerated. A discussion of the radiobiological reasons why this might be the case is outside the scope of this paper, but certainly causes us to question the validity of the EQD2 calculations in Table 3. The proof of any fractionation schedule can only be demonstrated by long term biochemical outcomes from carefully designed clinical trials.

We intend to follow this work with a pilot study of 2 fraction SBRT on the MR-Linac. Clinical implementation will require the availability of real-time plan adaptation and ideally a tracking strategy. Supplementary plan delivery verification work is needed to validate margins.

In conclusion, deliverable plans for MR-guided 1- and 2-fraction SBRT can be created for the MR-Linac, using dose constraints and objectives from HDR and SBRT clinical studies. Clinical validation of this work is planned.

## Appendix A Evaluation of the impact of bulk electron density assignment for MR-based UHF prostate treatment planning

For five patients of the ten included in the study, treatment plans generated using bulk relative electron density (ED) values assigned to the bones, CTV and external ROIs were recalculated using the CT HU-ED lookup table. Dose calculation settings were unchanged for the CT LUT calculation (see Table 4). Doses were exported from the treatment planning system and a voxel-wise comparison was performed using the gamma index with gamma criteria of 2% global dose difference and 2 mm distance to agreement. The fraction of gamma voxels with a gamma index < 1 was measured for voxels where the dose in the CT LUT calculation is above 10% the prescription dose.

For the 5 fraction treatment plans (reference dose 40 Gy), the gamma pass-rate mean and range was 97.0% (96.5 – 97.4%); for 2 fraction treatment plans (reference dose 27 Gy), 97.0% (96.5 – 97.3%) and for single fraction treatment plans (reference dose 21 Gy), 95.8% (94.2 – 96.9%).

Whilst the CT LUT dose is more accurate than the dose calculated with the assignment of bulk densities (due to the incorporation of ED heterogeneity), the use of bulk density assignment results in only small deviations in all of the plans assessed. The patient-specific bulk ED assignment strategy is considered sufficiently accurate for treatment planning in pelvic region due to the relatively low ED heterogeneity, in particular given the insensitivity of 7 MV x-ray attenuation to the range observed (ED 0.95 to 1.20).
