# Supplementary material for: Feasibility of MR-guided ultrahypofractionated radiotherapy in 5, 2 or 1 fractions for prostate cancer
Source: Clin Transl Radiat Oncol. 2020 Oct 27;26:1–7. doi: 10.1016/j.ctro.2020.10.005 (PMC7674276; doi:10.1016/j.ctro.2020.10.005)
Supplement: Supplementary data 1 [file mmc1.docx]

# Supplementary material

Table S1. Dose prescriptions, planning constraints and results for the UHF schemes investigated. PACE planning constraints (NCT01584258) were applied to 5 fraction plans, while for 2 and 1 fractions, constraints were derived from those reported in the HDR brachytherapy and SBRT literature (see Tables 1 & 2). Pass rate is the percentage of ten plans in which the clinical goal was satisfied.

| **Plan** | **ROI** | **Clinical goal** | | | **Unit** | **Median (Range)** | **Pass rate (%)** |
| --- | --- | --- | --- | --- | --- | --- | --- |
| CTV D95% ≥ 40 Gy and PTV D95% ≥ 36.25 Gy in 5 fractions | CTV_Prostate | D95% | ≥ | 40 | Gy | 40.0 (40.0 - 40.0) | 100 |
|  | PTV_Prostate | Max. dose | < | 48 | Gy | 44.0 (43.6 - 44.4) | 100 |
|  | PTV_Prostate | D2% | < | 42.8 | Gy | 42.6 (42.3 - 42.8) | 100 |
|  | PTV_Prostate | D95% | ≥ | 36.25 | Gy | 38.2 (37.7 - 38.5) | 100 |
|  | PTV_Prostate | V34.4Gy | ≥ | 98 | % | 100.0 (99.9 - 100.0) | 100 |
|  | Bladder | V37Gy | < | 5 | cc | 2.1 (1.1 - 5.0) | 100 |
|  | Bladder | V18.1Gy | < | 40 | % | 10.2 (2.3 - 21.9) | 100 |
|  | Rectum | V36Gy | < | 1 | cc | 0.5 (0.3 - 1.0) | 100 |
|  | Rectum | V29Gy | < | 20 | % | 5.1 (3.6 - 7.2) | 100 |
|  | Rectum | V18.1Gy | < | 50 | % | 22.7 (12.1 - 28.5) | 100 |
|  | PenileBulb | V29.5Gy | < | 50 | % | 0.0 (0.0 - 0.0) | 100 |
|  | Urethra | V42Gy | < | 50 | % | 10.1 (3.5 - 29.7) | 100 |
|  | Bowel | V30Gy | < | 1 | cc | 0.0 (0.0 - 0.0) | 100 |
|  | Bowel | V18.1Gy | < | 5 | cc | 0.0 (0.0 - 0.0) | 100 |
|  | FemoralJoint_L | V14.5Gy | < | 5 | % | 0.4 (0.0 - 0.9) | 100 |
|  | FemoralJoint_R | V14.5Gy | < | 5 | % | 0.1 (0.0 - 0.4) | 100 |
| GTV D95% ≥ 27 Gy and PTV D95% ≥ 24 Gy in 2 fractions | GTV_Prostate_Boost | Max. dose | < | 33.75 | Gy | 29.6 (29.4 - 30.1) | 100 |
|  | GTV_Prostate_Boost | D95% | ≥ | 27 | Gy | 27.1 (25.5 - 27.4) | 90 |
|  | PTV_Prostate | D2% | < | 29.7 | Gy | 28.5 (28.1 - 28.9) | 100 |
|  | PTV_Prostate | D95% | ≥ | 24 | Gy | 24.0 (22.5 - 24.3) | 60 |
|  | PTV_Prostate | D98% | ≥ | 22.8 | Gy | 22.9 (21.4 - 23.7) | 60 |
|  | PTV_Prostate | V22.8Gy | ≥ | 98 | % | 97.6 (93.1 – 99.7) | 60 |
|  | PTV_Prostate - GTV | Max. dose | < | 30 | Gy | 29.7 (29.2 - 30.0) | 100 |
|  | Bladder | V20.8Gy | < | 5 | cc | 4.2 (3.0 - 5.0) | 100 |
|  | Bladder | V14.6Gy | < | 15 | cc | 11.1 (7.4 - 14.4) | 100 |
|  | Rectum | V20.8Gy | < | 1 | cc | 0.8 (0.3 - 1.0) | 100 |
|  | Rectum | V17.6Gy | < | 4 | cc | 2.0 (1.2 - 2.8) | 100 |
|  | Rectum | V13Gy | < | 7 | cc | 4.9 (3.4 - 6.2) | 100 |
|  | Urethra | D10% | < | 27 | Gy | 26.7 (26.5 - 27.0) | 100 |
|  | FemoralJoint_L | V14Gy | < | 10 | cc | 0.0 (0.0 - 2.2) | 100 |
|  | FemoralJoint_R | V14Gy | < | 10 | cc | 0.0 (0.0 - 0.0) | 100 |
| GTV D95% ≥ 21 Gy and PTV D95% ≥ 19 Gy in 1 fraction | GTV_Prostate_Boost | Max. dose | < | 26.25 | Gy | 23.3 (22.9 - 24.0) | 100 |
|  | GTV_Prostate_Boost | D95% | ≥ | 21 | Gy | 21.1 (19.7 - 21.6) | 90 |
|  | GTV_Prostate_Boost | D99% | ≥ | 19.95 | Gy | 20.7 (18.9 - 21.3) | 90 |
|  | PTV_Prostate | D95% | ≥ | 19 | Gy | 19.0 (18.3 - 19.2) | 70 |
|  | PTV_Prostate | D99% | ≥ | 18.05 | Gy | 18.1 (16.6 - 18.4) | 60 |
|  | PTV_Prostate | V18.05Gy | ≥ | 99 | % | 98.5 (95.6 – 99.6) | 60 |
|  | PTV_Prostate - GTV | Max. dose | < | 23.75 | Gy | 23.3 (22.8 - 23.6) | 100 |
|  | Bladder | D50% | < | 12 | Gy | 1.0 (0.5 - 4.2) | 100 |
|  | Rectum | D0.04cc | < | 19 | Gy | 18.4 (18.0 - 19.0) | 100 |
|  | Rectum | D2cc | < | 15 | Gy | 14.8 (12.8 - 15.0) | 100 |
|  | Rectum | D50% | < | 12 | Gy | 3.9 (0.8 - 7.1) | 100 |
|  | Urethra | Max. dose | < | 22.8 | Gy | 21.2 (20.7 - 21.8) | 100 |
|  | Urethra | D10% | < | 21 | Gy | 20.7 (20.3 - 21.0) | 100 |
